# Frequencies of *CYP2D6* genetic polymorphisms in Arab populations

**DOI:** 10.1186/s40246-022-00378-z

**Published:** 2022-02-05

**Authors:** Mousa Alali, Wouroud Ismail Al-khalil, Sara Rijjal, Lana Al-Salhi, Maher Saifo, Lama A. Youssef

**Affiliations:** 1 Department of Oncology, Albairouni University Hospital, Faculty of Medicine, Damascus University, Damascus, Syrian Arab Republic; 2 Program of Clinical and Hospital Pharmacy, Department of Pharmaceutics and Pharmaceutical Technology, Faculty of Pharmacy, Damascus University, Mezzeh Autostrad, Damascus, Syrian Arab Republic; 3 Faculty of Pharmacy, International University for Science and Technology (IUST), Ghabagheb, Daraa Syrian Arab Republic; 4 National Commission for Biotechnology (NCBT), Damascus, Syrian Arab Republic

**Keywords:** *CYP2D6*, Duplications, Allele frequency, Single nucleotide polymorphisms, Genotype, Arabs, Middle East, North Africa

## Abstract

CYP2D6 is a key drug-metabolizing enzyme implicated in the biotransformation of approximately 25% of currently prescribed drugs. Interindividual and interethnic differences in CYP2D6 enzymatic activity, and hence variability in substrate drug efficacy and safety, are attributed to a highly polymorphic corresponding gene. This study aims at reviewing the frequencies of the most clinically relevant *CYP2D6* alleles in the Arabs countries. Articles published before May 2021 that reported *CYP2D6* genotype and allelic frequencies in the Arab populations of the Middle East and North Africa (MENA) region were retrieved from PubMed and Google Scholar databases. This review included 15 original articles encompassing 2737 individuals from 11 countries of the 22 members of the League of Arab States. Active *CYP2D6* gene duplications reached the highest frequencies of 28.3% and 10.4% in Algeria and Saudi Arabia, respectively, and lowest in Egypt (2.41%) and Palestine (4.9%). Frequencies of the loss-of-function allele CYP2D6*4 ranged from 3.5% in Saudi Arabia to 18.8% in Egypt. The disparity in frequencies of the reduced-function CYP2D6*10 allele was perceptible, with the highest frequency reported in Jordan (14.8%) and the lowest in neighboring Palestine (2%), and in Algeria (0%). The reduced-function allele CYP2D6*41 was more prevalent in the Arabian Peninsula countries; Saudi Arabia (18.4%) and the United Arab Emirates (15.2%), in comparison with the Northern Arab-Levantine Syria (9.7%) and Algeria (8.3%). Our study demonstrates heterogeneity of *CYP2D6* alleles among Arab populations. The incongruities of the frequencies of alleles in neighboring countries with similar demographic composition emphasize the necessity for harmonizing criteria of genotype assignment and conducting comprehensive studies on larger MENA Arab populations to determine their *CYP2D6* allelic makeup and improve therapeutic outcomes of CYP2D6- metabolized drugs.

## Background

Interindividual variability in drug response, affecting both drug efficacy and safety, is perceived as a major challenge in clinical practice. Intrinsic factors (age, gender, ethnicity, pregnancy, lactation, and comorbidities), as well as extrinsic ones (environment, smoking, nutrition, alcohol consumption, and drug interactions), can influence the response to therapeutic drugs [1]. The last few decades have witnessed a mounting interest in the significance of genetic variations in genes encoding key drug transporters, metabolizing enzymes, and targets, owing to their explanatory contribution of approximately 20–30% of the variability in drug response [2].

Strikingly, polymorphic enzymes, mainly members of the cytochrome P450 superfamily, metabolize 60–80% of all prescribed drugs [3]. Cytochrome P450 2D6 (CYP2D6) constitutes only 1–5% of total hepatic CYPs content; nevertheless, it is involved in metabolizing approximately 25% of currently available drugs; such as tricyclic antidepressants, selective serotonin reuptake inhibitors, antipsychotics, opioids (e.g., codeine, and tramadol), antiarrhythmics, β-blockers, antineoplastic agents (e.g., tamoxifen and gefitinib), and a variety of other drugs [3, 4].

CYP2D6 is encoded by a highly polymorphic gene that has over 140 allelic variants characterized to date [5]. The *CYP2D6* gene is located on the long arm of chromosome 22 (22q13.2) in a gene cluster that also comprises two highly homologous pseudogenes, *CYP2D7* and *CYP2D8*. It consists of nine exons and contains 4382 base pairs that code for a 497-amino acid protein [6]. The *CYP2D6* alleles are classified into; null alleles (e.g., *3, *4, *5, *6, and *4xN) that cause ablation or absence of enzymatic activity, reduced-function alleles (e.g., *9, *10, *17, *29, and *41) that result in decreased functional products, normal function alleles (e.g., *1, *2, *33, *35, *17 × *2*, *29 × *2*, and *41 × *2*) that possess normal activity, and increased function alleles (*1xN, *2xN, *35 × *2*, and *45 × *2*) with higher CYP2D6 activity. In addition, there is a considerable number of alleles whose function is still unknown (e.g., *58, *73, *74, and *85), or uncertain (e.g., *22, *23, *37, and *43) [5, 7].

Due to the complexity of the *CYP2D6* gene and allelic combinations, translating *CYP2D6* genotype into phenotype is quite challenging. The activity score (AS) system suggested by Gaedigk et al. (2008) has been adopted and standardized by the Clinical Pharmacogenetics Implementation Consortium (CPIC) and the Dutch Pharmacogenetics Working Group (DPWG) [7, 8]. In order to facilitate the assignment of an individual’s phenotype, each *CYP2D6* allele is assigned a value from zero to one that reflects its activity, which in turn is used to allocate four distinctive phenotypes based on the individuals’ allelic combination in their diplotype. Poor metabolizers (PMs, AS = 0) exhibit an absolute lack of CYP2D6 activity, whereas intermediate metabolizers (IMs, 0.25 ≤ AS ≤ 1) have reduced CYP2D6 metabolic capacity relative to that of normal metabolizers (NMs, 1.25 ≤ AS ≤ 2.25). *CYP2D6* ultrarapid metabolizers (UMs, AS > 2.25) demonstrate a higher CYP2D6 activity than NMs, and subsequently rapid metabolism of CYP2D6 substrates [7].

The frequencies of *CYP2D6* alleles vary significantly between ethnic groups and geographical regions, resulting in interethnic variability of predicted phenotypes [9]. For instance, CYP2D6*4 (defined by rs3892097; NC_000022.11:g.42128945C>T) is predominant in Europeans, signifying a relatively high prevalence of PM phenotype among this population. On the other hand, IM phenotype is more commonly observed among East Asians, in part due to the highest prevalence (~ 41%) of the reduced-function CYP2D6*10 (defined by rs1065852; NC_000022.11:g.42130692G>A) allele. In contrast, Middle Easterners are characterized by a higher frequency of the CYP2D6*1 × *N* and CYP2D6*2 × *N* duplication alleles and the reduced-function CYP2D6*41 (defined by rs28371725; NC_000022.11:g.42127803C>T) allele. Furthermore, the reduced-function CYP2D6*17 (defined by rs28371706; NC_000022.11:g.42129770G>A) and CYP2D6*29 (defined by rs61736512; NC_000022.11:g.42129132C>T + rs59421388; NC_000022.11:g.42127608C>T) alleles are more frequent among African and African American populations [10, 11]. Owing to the critical impact of *CYP2D6* genotypes on enzymatic activity, hence substrate drug metabolism, it is of great interest to determine the frequencies of *CYP2D6* alleles in different populations to improve genotype-guided drug response predictions. However, information on the genotypes and the frequencies of *CYP2D6* alleles in Arab populations is scarce. Here, we review the published data on the prevalence of *CYP2D6* alleles among Arab populations of the MENA countries defined as members of the League of Arab States in Western Asia, North Africa, and the Horn of Africa.

## Methods

A literature search of PubMed and Google Scholar databases was conducted using the following keywords; *"CYP2D6*" and "allele" or "frequency" or "polymorphism" or "genotype" or "gene duplications," and the nationalities or the 22 Arab country names (Algeria, Bahrain, Comoros, Djibouti, Egypt, Iraq, Jordan, Kuwait, Lebanon, Libya, Mauritania, Morocco, Oman, Palestine, Qatar, Saudi Arabia, Somalia, Sudan, Syria, Tunisia, United Arab Emirates "UAE", or Yemen). All original articles published in English before May 2021 were included. Moreover, a supplementary manual search of the reference lists of included studies and relevant review articles was performed to identify any article not retrieved from searching databases.

Studies were excluded if: they did not report the exact frequency of the concerned *CYP2D6* alleles and only referred to them as mutant alleles, they were confined to CYP2D6 phenotypes regardless of genotypes, the genotyping approach is equivocal, or the genotyping details that support the resulting frequencies are not provided. Reviews, case reports, or studies conducted in Arab countries but on non-Arabs were also excluded. Two independent reviewers screened titles*,* abstracts, and full-text articles. The following information was extracted from each study: the Arab country, number and type of each sample (healthy subjects or patients), the genotyping method, and the frequencies of investigated alleles and CYP2D6 phenotypes. In case the assignment of phenotypes did not follow the updated AS system, we re-estimated the frequency of different CYP2D6 phenotypes where applicable [7]. Furthermore, CYP2D6*1 was not calculated in the present review due to its default assignment and imprecise frequency, which varies according to the number of single nucleotide polymorphisms (SNPs) identified in each study.

## Results

The literature search revealed 15 studies, encompassing 2737 individuals, that met the inclusion criteria. Four studies were conducted in Egypt, two in Saudi Arabia, one study each in Iraq, Jordan, Lebanon, Morocco, Tunisia, and the UAE. Additionally, international studies that included Arab populations were also included. Unpublished data of our own on the frequencies of *CYP2D6* alleles in Syrian breast cancer patients were also included. In our study, genotyping was performed using targeted standard sequencing of specific polymerase chain reaction (PCR) products containing the gene loci of three SNPs 100C>T, 1847G>A, and 2989G>A. Subsequently, star alleles including CYP2D6*4, CYP2D6*10, and CYP2D6*41 were assigned based on each individual’s haplotype.

Our search revealed the absence of published studies on the frequencies of *CYP2D6* alleles in 10 of 22 Arab countries including Bahrain, Comoros, Djibouti, Kuwait, Libya, Mauritania, Oman, Qatar, Somalia, and Yemen. Studies’ objectives varied and were concerned with investigating the frequency of *CYP2D6* genetic polymorphisms in a healthy cohort of subjects, determining the influence of *CYP2D6* genotype on the therapeutic outcomes of CYP2D6-metabolized drugs, and/or exploring a plausible relationship between *CYP2D6* genotype and susceptibility to some diseases. Genotyping was performed in the majority of the included studies by polymerase chain reaction-restriction fragment length polymorphism (PCR–RFLP) analysis [12–19]. Standard sequencing, real-time PCR, and long PCR were the applied genotyping methods in a few studies [20–25, and our unpublished data]. The frequencies of *CYP2D6* alleles and gene duplications in various Arab countries are summarized in Tables 1 and 2, respectively.

**Table 1:** Frequencies of *CYP2D6* alleles in the Arab countries

Country/References	No. of individuals	Subject type	Genotyping method	Alleles’ activity							
				Normal	Reduced			Non-functional			
				*2	*10	*17	*41	*3	*4	*5	*6
Algeria (Mozabite) [20]	30	–	Long PCR	28.3	0	8.3	8.3	0	11.7	3.3	0
Egypt [12]	308	Healthy fertile + infertile men	RFLP analysis	–	–	–	–	–	25.6	–	–
Egypt [13]	29	Healthy subjects	RFLP analysis	–	–	–	–	–	18.1	–	–
	40	Acute OP^a^ intoxicated patients									
Egypt [14]	29	Healthy subjects	RFLP analysis	–	–	–	–	–	22.0	–	–
	30	Chronic OP^a^ exposed patients									
Egypt [21]	145	Healthy subjects	TaqMan	31.3	3.4	–	15.1	–	9.6	2.0	–
Iraq [15]	250	ACS^b^ patients	RFLP analysis	7.6	13.4	9.2	–	–	6.8	–	–
Jordan [16]	192	Healthy subjects	RFLP analysis	–	14.8	8.3	–	–	12.8	–	–
Lebanon [22]	111	Breast cancer patients	Real-time PCR	–	–	–	12.1	0.9	15.9	–	–
Morocco [17]	200	Healthy subjects	RFLP analysis	–	8.5	–	–	11.13	10.1	–	–
	200	Breast cancer patients									
Palestine [20]	51	–	Long PCR	27.5	2.0	2.0	12.7	0	7.8	1.0	2.0
Saudi Arabia [23]	192	Healthy subjects	Sequencing	–	–	–	18.4	0	–	–	0
Saudi Arabia [18]	101	Healthy subjects	RFLP analysis	–	3.0	3.0	–	–	3.5	1.0	–
Syria [24]	51	Healthy subjects	Long PCR	30.39	2.94	0	–	0	9.8	0.98	0.98
Syria unpublished data	97	Breast cancer patients	Sequencing	–	7.2	–	9.28	–	7.2	–	–
Tunisia [19]	300	Breast cancer patients	RFLP analysis	–	–	–	–	–	13.39	–	–
	230	Healthy subjects									
UAE [25]	101	Psychiatric patients	Sequencing	12.2	3.3	2.5	15.2	0	9.0	–	0
	50	Healthy subjects									

^a^OP: organophosphate, ^b^ACS: acute coronary syndrome

**Table 2:** Frequencies of* CYP2D6* gene duplications in Arab countries

Country/References	Duplicated alleles					All duplications combined
	*4xN	*10xN	*41xN	*1xN	*2xN	*xN*
Algeria (Mozabite) [20]	0	0	0	0	28.3	28.3
Egypt [21]	0	(0–0.35)^a^	(0–1.04)^a^	(2.41–3.8)^a^		3.8
Jordan [16]	–	–	–	–	6.75	6.75
Lebanon [22]	–	–	–	–	–	9.45
Palestine [20]	0	0	0	0	4.9	4.9
Saudi Arabia [18]	–	–	–	–	10.4	10.4
Syria [24]	0	–	–	3.92	3.92	7.84
UAE [25]	–	0.3	–	1.6	4.3	6.2

^a^Mutawi et al. identified 4 subjects with *2/*41 (*3N*) and *2/*10 (*3N*) genotypes without discrimination of which allele has been duplicated

### Frequencies of *CYP2D6* null alleles

**Fig. 1:**
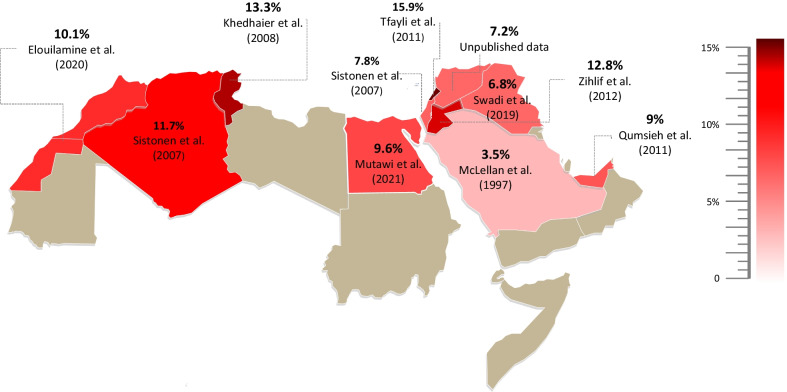
Arab map of the most clinically important *CYP2D6* null allele CYP2D6*4. Frequencies ranged between 15.9% in Lebanon (dark red) and 3.5% in Saudi Arabia (light red)

CYP2D6*4 was the most studied null allele with frequencies showing great variability between Arabs (Fig. 1). The highest prevalence was observed in Egypt with an average frequency of (18.8%), whereas the lowest frequency was reported in Saudi Arabia (3.5%).

In North Africa, CYP2D6*3 allele was reported at a high frequency of (11.13%) in Morocco, and to the contrary, was absent in neighboring Algeria. It presented at substantially low frequency or was absent in the Levant; Lebanon (0.9%), Palestine (0%) and Syria (0%), and the Arabian Peninsula; Saudi Arabia (0%) and UAE (0%). *CYP2D6* deletion variant CYP2D6*5 was investigated only in five countries that revealed the paucity of this allele among Arabs with a frequency ranging from 0.98% in Syrians to 3.3% in Algerians. Similarly, CYP2D6*6 frequency pivoted around 0–2%. Only one study in Saudi Arabia reported a frequency of 0.3% of the scarce CYP2D6*14 null allele.

### Frequencies of *CYP2D6* reduced-function alleles

**Fig. 2:**
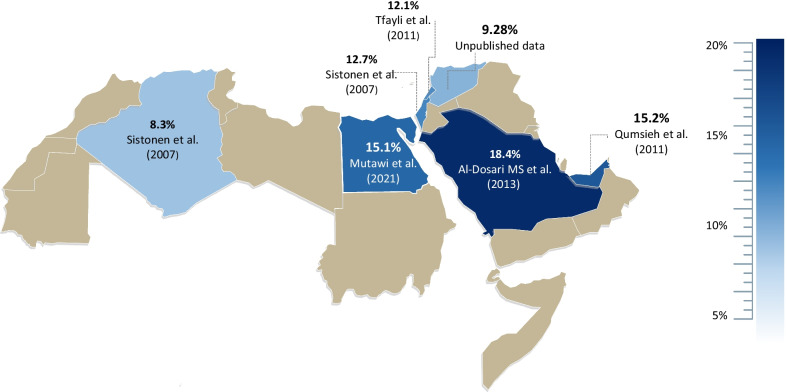
Arab map of CYP2D6*41 frequencies. A South-to-North CYP2D6*41 gradient frequencies were observed, as the highest frequency was found in the Arabian Peninsula Saudi Arabia (18.4%, dark blue) and lowest in North Levantine Syria (9.28%, light blue), and a similar trend of West-to-East gradient was exemplified by a higher frequency in Egypt (15.1%, dark blue) and lower prevalence in Algeria (8.3%, light blue)

Amongst the reduced-function alleles, CYP2D6*41 ranked as the most prevalent allele with a clear South-to-North gradient ranging from the lowest in the Levantines (9.28% in Syrians, 12.1% in Lebanese, and 12.7% in Palestinians) to 15.2% in the UAE and 18.4% in Saudi Arabia, where the highest frequency was reported among Arabs. Additionally, a West-to-East gradient was evident in North African countries; as a relatively lower frequency was observed in Algeria (8.3%) in comparison to Egypt (15.1%) (Fig. 2).

**Fig. 3:**
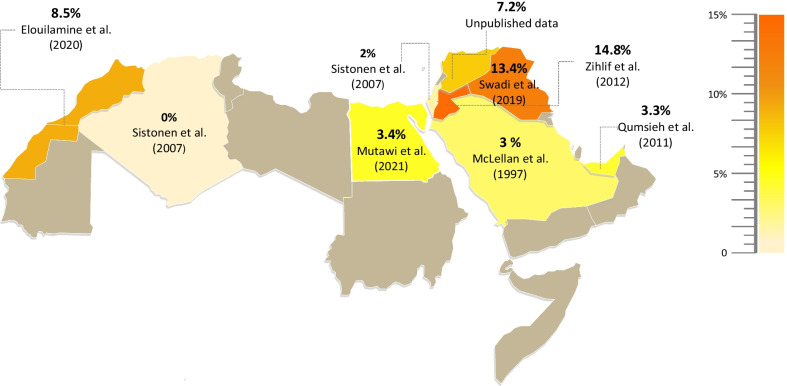
Arab map of CYP2D6*10 frequencies. The highest frequencies were in Jordan (14.8%, orange) and lowest were in Palestine and Algeria (2% and 0%, respectively, light yellow)

CYP2D6*10 was the most investigated reduced-function allele. It was reported in Jordan and Iraq at frequencies of 14.8% and 13.4%, respectively. The prevalence was much lower in Palestine (2%), Egypt (3.4%), UAE (3.3%), and Saudi Arabia (3%). CYP2D6*10 was absent in Algeria; to the contrary of neighboring Morocco where a substantially higher frequency of 8.5% was reported (Fig. 3). The prevalence of CYP2D6*17 was relatively low in the Arab countries, except for Iraq, Algeria, and Jordan, in which similar frequencies of 9.2%, 8.3%, and 8.3% were reported, respectively. Other rare alleles such as CYP2D6*29 were only screened in Saudi Arabia (2.9%), UAE (1.6%), Algeria (0%), and Palestine (0%).

### Normal function CYP2D6*2 allele and *CYP2D6* gene duplications

**Fig. 4:**
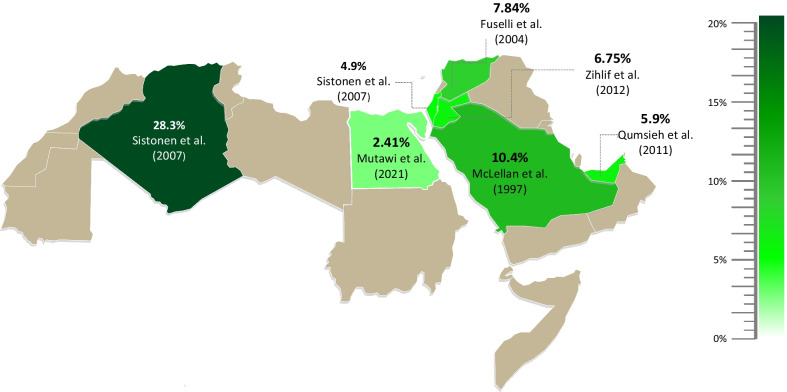
Arab map of active *CYP2D6* gene duplications and multiplications (CYP2D6*1xN and CYP2D6*2xN), which were most common in Algeria, and Saudi Arabia (28.3% and 10.4%, respectively, dark green)

The frequency of the functional CYP2D6*2 allele ranged between 7.6% and 31.3% among Arabs. The majority of gene duplication events were observed for the functional CYP2D6*2xN allele. Active gene duplications were most prevalent in Algeria with a frequency of 28.3%, and Saudi Arabia (10.4%) (Table 2, Fig. 4). Duplications of other reduced-function and nonfunctional alleles were only screened in a few studies and none of them reported CYP2D6*4xN gene duplication. CYP2D6*10xN was reported at a low frequency in UAE (0.3%). Mutawi et al. (2021) identified three subjects with the *2/*41 (*3N*) genotype and one subject with *2/*10 (*3N*) genotype in Egyptians. Due to the inability to discriminate which allele has been duplicated, CYP2D6*10xN and CYP2D6*41xN frequencies in Egypt cannot be precisely estimated and are expected to range between 0–0.35% and 0–1.04%, respectively (Table 2).

### Frequencies of CYP2D6 phenotypes

**Fig. 5:**
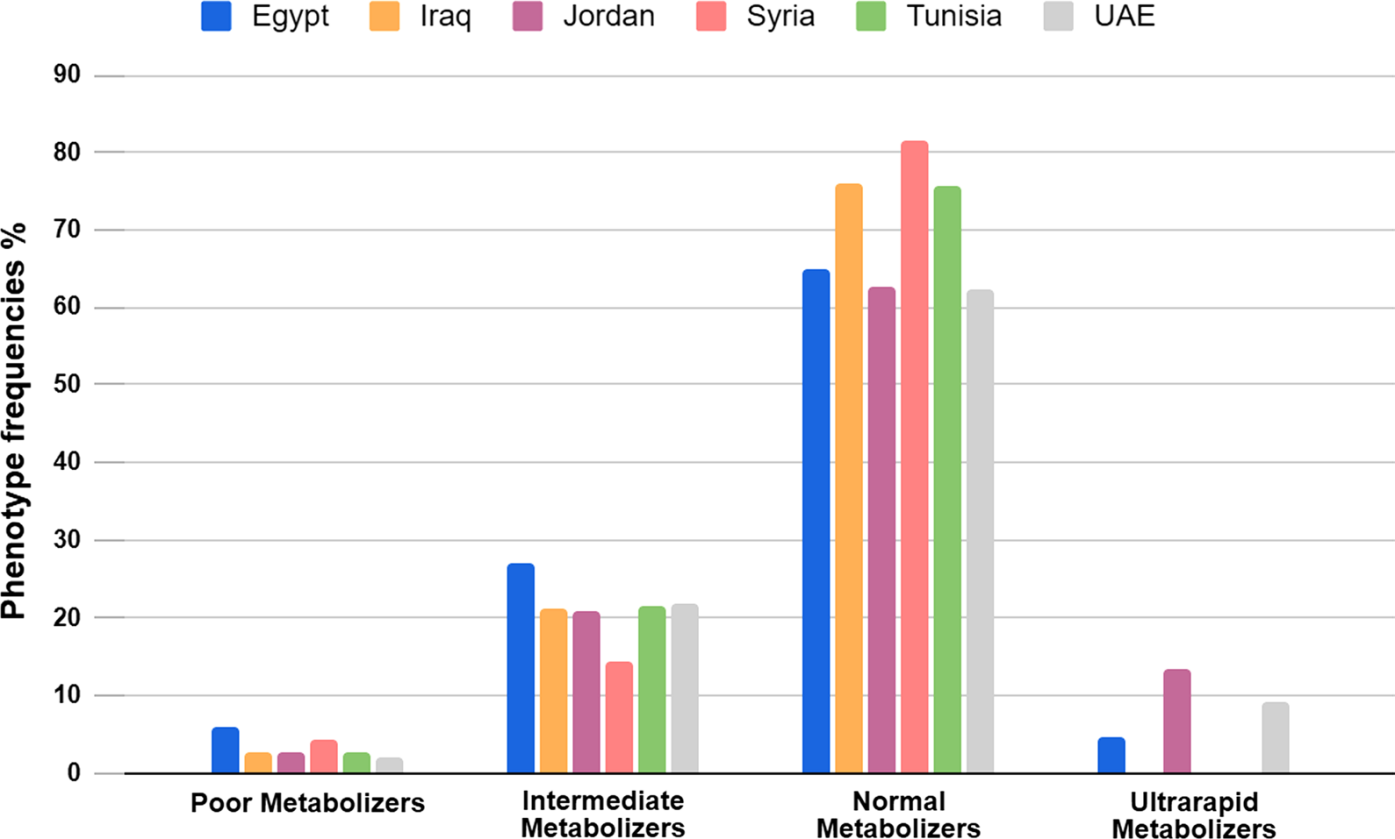
The frequencies of CYP2D6 phenotypes in different Arab countries. NM phenotype is the most prevalent phenotype among Arabs followed by IM, UM and lastly PM

The frequencies of the different *CYP2D6* genotypes and their corresponding phenotypes were available for only six countries including Egypt, Iraq, Jordan, Syria, Tunisia, and UAE (Table 3, Fig. 5). The average frequencies of CYP2D6 phenotypes among Egyptians were estimated based on data derived from four studies. NM phenotype was the predominant phenotype among Arabs (70.53%). In contrast, the percentage of individuals with PM phenotype was found to be relatively low in Arabs (3.39%) with the highest frequency reported in Egypt (6.08%) and lowest in UAE (2%). IM phenotype was most prevalent in Egypt (27.11%), and lowest in Syria (14.4%). Moreover, UM phenotype was presented at a relatively high frequency among Arabs (9.2%), with the highest frequency reported in Jordanians (13.5%).

**Table 3:** Frequencies of CYP2D6 phenotypes in Arab countries

Country	Poor Metabolizers	Intermediate Metabolizers	Normal Metabolizers	Ultrarapid Metabolizers	References
Egypt	6.08	27.11	65.08	4.83	[12–14, 21]
Iraq	2.8	21.2	76	–	[15]
Jordan	2.6	21.1	62.5	13.5	[16]
Syria	4.2	14.4	81.5	–	Unpublished data
Tunisia	2.64	21.51	75.85	–	[19]
UAE	2	21.85	62.25	9.27	[25]
Arab countries (average)	3.39	21.2	70.53	9.2

## Discussion

Interethnic variability in individuals' metabolic capacity due to genetic differences has been well-established. The clinical relevance of this phenomenon is mirrored by the apparent ethnic and regional variability in drug response and susceptibility to some diseases. Accordingly, a plethora of pharmacogenetic studies has contributed to gaining insights into the genetic basis of drug response variabilities in various global populations.

CYP2D6 has gained enormous attention due to its key role in metabolizing a significant proportion of widely used drugs. Historically, phenotype studies relying on metabolic assessments using CYP2D6 probe drugs, such as debrisoquine and sparteine, have revealed a highly interindividual variability in metabolic activity ranging from complete deficiency to excessive activity [26, 27]. This variability is attributed in part to the highly polymorphic nature of the *CYP2D6* gene, whose allelic profile varies substantially between ethnicities.

Despite the abundance of studies that have investigated the frequencies of *CYP2D6* alleles worldwide, only a few have evaluated the distribution of *CYP2D6* alleles in Arabs. In this review, we highlight and consolidate the available information to date regarding the prevalence of *CYP2D6* alleles across Arab countries. Moreover, we address the gaps in our knowledge regarding the genetic background of these unique populations. The Arab world stretches over Western Asia, North Africa, and the Horn of Africa and comprises 22 countries with a total population of over 436 million people in 2020, thus constituting approximately 5.6% of the total world's population [28].

The observed differences in the frequencies of *CYP2D6* alleles in the Arab populations could be attributed to demographic as well as technical factors. Firstly, the MENA region has been historically a "melting pot" for human migrations, which resulted in a remarkable ethnic, cultural, and genetic diversity. Secondly, the included studies used various genotyping methods, and more than half of them utilized PCR–RFLP analysis for detecting *CYP2D6* alleles. In general, no such ideal genotyping method exists [29], and all the applied methods are widely used and have proved high reliability in *CYP2D6* genotyping [30]. However, each method has its advantages and limitations. For instance, PCR–RFLP analysis is inexpensive and does not require complex instrumentation; however, it is a very laborious strategy and consists of several sequential and mostly nonautomated steps [29]. Moreover, PCR–RFLP-based-genotyping is not always precise when there is more than one nucleotide variation in the restriction enzyme recognition site [31]. Furthermore, genetic analysis of the *CYP2D6* gene is quite challenging. Several limitations have been widely described such as the presence of highly homologous *CYP2D7* and *CYP2D8* pseudogenes within the gene locus, the occurrence of copy number variations (CNVs), and structural variants that give rise to *CYP2D7*-*2D6* hybrids in single entities or tandem arrangements [32]. On the other hand, the relatively small sample size in some studies, such as those in Algeria (n = 30) and Palestine (n = 51), may be inadequate to reflect the actual allelic frequencies in these countries. Another important point to discuss is the differences in the characteristics of the evaluated subjects (patients versus healthy volunteers). Generally, CYP2D6 is not directly related to any disease, and the average allelic frequencies for each ethnic group provided by the Pharmacogenomics Knowledge Base (PharmGKB) were calculated based on studies of both healthy and patient individuals [33, 34]. However, CYP2D6 is involved in metabolizing and detoxifying numerous xenobiotics [35] and some of the included studies have proved a linkage between *CYP2D6* genetic polymorphisms and susceptibility to diseases [12–14, 17, 19]. Therefore, in the absence of profound evidence, we here reported the frequency of each *CYP2D6* allele in the overall population rather than only healthy subjects. Nevertheless, we cannot exclude the health state of the subjects as a source of variability.

Our current analysis of UM phenotype estimation in Arabs (9.2%) corresponds to the results reported by a previous review by LLerena et al. (2014) in which they demonstrated that UMs are most prevalent in the Middle Eastern population with a frequency of 10.45% [10]. Our results are also in line with the frequency reported by the PharmGKB CYP2D6 reference material for Near Easterners (9.47%) [34]. Middle Eastern Arab populations exhibited higher frequencies of CYP2D6*1xN and CYP2D6*2xN duplications (2.41–10.4%) than those reported in Americans (3.47%), Europeans (1.97%), Central/South Asians (1.51%), and East Asians (0.79%) [34]. Remarkably, the highest frequency (28.3%) of CYP2D6*2xN duplications in Algerians is comparable to that of Ethiopians (29%) [36]. Nevertheless, the main limitation of the Algerian data is the small size of the study cohort (n = 30). Moreover, the study subjects were only from the Mozabites and may not be representative of the entire Algerian population, hence, the estimated frequency should be interpreted with caution. Arabs besides Ethiopians are characterized by the highest prevalence of active *CYP2D6* gene duplications. Ingelman-Sundberg M (2005) suggested an evolutionary basis of this phenomenon and attributed it to the CYP2D6-mediated detoxifying of some constituents found in the local diets, especially alkaloids plants. Historically, 5000–10000 years ago, during periods of starvation, Ethiopians and Middle Easterners who harbored multiple functional *CYP2D6* alleles, such as UMs, gained a natural selection advantage over individuals with lower metabolizing capacity (e.g., NMs, IMs, and PMs). Consequently, an expansion of UMs' sub-population occurred in North East Africa and the Middle East [37].

In terms of non-functional alleles, the highly investigated CYP2D6*4 allele occurred with considerably lower frequencies across Arab populations compared to Europeans (18.54%), but higher than the reported frequencies in Sub-Saharan Africa (3.38%) and East Asian countries (0.54%) [34]. CYP2D6*4 frequencies varied between Arab countries, with a noticeable decrease in Saudi Arabians (3.5%), which can be interpreted by the previous presumption that food consumed by this population has resulted in selection pressure in CYP2D6 metabolic activity favoring carriers of *CYP2D6* alleles without deleterious mutations [18]. Furthermore, Saudi Arabians are expected to have Afro-Asian influence, which resulted in a decrease in CYP2D6*4 frequency [11]. The frequency of *CYP2D6* gene deletion (CYP2D6*5) was substantially low among Arabs. However, Algerians exhibited a relatively high frequency (3.3%) comparable to that in Central Europe (3%) but lower than that observed among Sub-Saharan African populations (5.15%) [34, 38]. The CYP2D6*3 null allele was almost absent in Arab countries except for Moroccans with an intriguingly high frequency of (11.13%), and thus exceeding by far frequencies in Europeans (1.59%) [34].

Globally, CYP2D6*41 is reported to be most common in Middle Easterners [10, 39]. Our comparative analysis has confirmed this observation, as CYP2D6*41 was the most predominant reduced-function allele with a relatively higher frequency in the Arabian Peninsula in comparison with Central/South Asians (12.3%), Sub-Saharan Africans (11.47%), Americans (2.33%), and East Asians (2.27%) [34]. The South-to-North gradient observed in our study is further supported by the documented decreased frequencies in Iran (8.71%) and European countries (9.24%) [34, 40]. Conversely, the frequency of CYP2D6*10 allele showed mixed patterns between Arab populations with no clear gradient, but still at much lower prevalence (0–14.8%) than frequencies reported in Asians (43.5%) [34].

The resemblances of the demographic composition of the five Arab countries (Syria, Lebanon, Palestine, Jordan, and Iraq) that constitute the Levant and Mesopotamia regions suggest similarities in their genetic structure. However, the conflicting frequencies of CYP2D6*10 allele in Iraq (13.4%), Jordan (14.8%), Syria (7.2%), and Palestine (2%) are puzzling. Jordan's population includes Syrians and Palestinians. Prominently, Palestinians and their descendants blended into the Jordanian society and are estimated to constitute more than half of the population of Jordan. These variabilities in allelic frequencies may be due to how alleles are assigned, which can cause discrepancies in determining genotypes and estimating frequencies. Some of the included studies in this review reported allelic frequency in terms of a single variant detected, which might not always reflect the actual frequency of alleles that are defined by several variants. The CYP2D6*10 allele, for instance, is defined by the alteration 100C>T, which is also present in 22 other alleles and most notably in the non-functional CYP2D6*4 allele [5]. Thus, assigning only the 100C>T variant to the CYP2D6*10 allele without ruling out other haplotypes carrying the 100C>T variant may result in overestimating the frequency of CYP2D6*10 [32]. In fact, this observation may explain the aforementioned inconsistent frequencies between Levantines, as Jordanians, as well as Iraqis, assigned the frequency of the variant 100C>T to CYP2D6*10, whereas the documented frequencies in both Syrian and Palestinian populations reflect the actual haplotype frequency. Therefore, we speculate that CYP2D6*10 allelic frequency among Iraqis and Jordanians is comparable to that of Syrians, as Palestinian data is based on a relatively small study cohort (n = 50). Additionally, the function-altering 2989G>A variant is usually used to identify CYP2D6*41. However, this variant is also not unique to CYP2D6*41 as other six rare alleles harbor this SNP as well. For example, CYP2D6*69 null allele carries the defining alleles of both CYP2D6*41 and CYP2D6*10 [41]. Consequently, CYP2D6*41 frequency is also prone to misinterpretation and overestimation. This is exemplified by our observation of a frequency of 11.86% for the variant 2989G>A in Syrians; however, the frequency of the CYP2D6*41 haplotype was found to be (9.28%), as CYP2D6*69 allele was relatively high in the Syrian population (2.58%) (Our unpublished data).

As expected, CYP2D6*17 presented at lower frequencies in Arabs (0–9.2%) when compared to Africans (19.29%), but with the exception of Syrians’ (0%), was still at a higher prevalence than that reported in the Europeans (0.39%) and South/Central Asians (0.07%) [34]. Collectively, the intermediate metabolizer phenotype in Arabs is mainly attributed to the presence of the reduced-function CYP2D6*41 allele and a lesser extent the CYP2D6*10 allele.

The geographic proximity and demographic similarities resulting from trade, wars, marriages, and historic migration flow suggest that the Arab Levantines (Syrians, Lebanese, Palestinians, and Jordanians) and Mesopotamians (Syrians and Iraqis) are closely related to other Near Eastern populations such as Iranians and Turks. Despite the discrepancy in the frequencies of the null CYP2D6*4 allele (7.2% to 15.9%) in the Levant and Mesopotamia, the average frequency of 10.05% was in harmony with the average frequency in Turkey (13.8%) and Iran (10.3%) [34]. The scarcity of the other null alleles (*3, *5, and *6) was also comparable between the Levant and Iran and Turkey. Similarly, the frequencies of the reduced-function CYP2D6*41 allele in Turkey (14%) and Iran (7.9%) were comparable to those reported in Syria (9.28%), Lebanon (12.1%), and Palestine (12.7%) [40, 42]. Furthermore, due to the inconsistency in assigning the CYP2D6*10 allele, a similar intra-ethnicity disparity of its estimated frequencies was evident in Iran (from 3 to 9%) and Turkey (from 6 to 14.5%). [40, 43–45].

In our analysis, we found that Individuals with normal metabolic capacity (NMs) constitute the majority of Arab populations. The frequency of NMs in Arabs (70.53%) was higher than that of Europeans (51.05%), East Asians (51.91%), and Americans (63.6%) [34]. As expected, PMs accounted for only a small percentage of Arabs (3.39%), which was higher than that of East Asians (0.86%), Sub-Saharan Africans (1.53%), and Americans (2.18%), but lower than that of Europeans (6.47%). This can be attributed to the highest prevalence of the null CYP2D6*4 allele among European populations (18.54%) [34]. However, our analysis has major limitations. Firstly, we cannot extrapolate the precise distribution of CYP2D6 phenotypes among Arabs based on the limited data available for only six countries. Multiple studies reported only the frequency of *CYP2D6* alleles without referring to the genotypes, and hence the metabolic phenotypes of the studied populations could not be determined. Furthermore, genotyping only a small set of *CYP2D6* alleles can considerably affect the resultant predicted phenotypes. For instance, a higher frequency of NMs was observed in studies that did not genotype a large number of *CYP2D6* allelic variants or did not estimate the frequency of gene duplications such as studies conducted in Tunisia, Iraq, and Syria (75.85%, 76%, and 81.5%, respectively). On the contrary, Mutawi et al. (2021) and Qumsieh et al. (2011) reported lower frequencies of NMs in both Egypt and UAE (67.6% and 62.25%, respectively), which mirror a more comprehensive coverage of *CYP2D6* alleles. It will be indispensable for future studies to report the various observed genotypes along with the AS and predicted phenotypes.

Due to the complexity of the *CYP2D6* gene locus, the presence of CNVs, the enormous number of identified SNPs, and the fact that some SNPs exist in multiple alleles, it is extremely challenging to unequivocally determine the exact individual’s *CYP2D6* genotype [32]. Many commercially available platforms assign the highest probability of an individual's genotype by identifying the most clinically important SNPs. However, the presence of rare variants, as illustrated by the CYP2D6*69 allele, should not be trivialized. Furthermore, researchers should be cautious and capable of distinguishing between the frequency of the variant and that of the haplotype (star allele), unless the identified SNP is unique to a particular allele such as 1847G>A (CYP2D6*4).

The clinical implications of CYP2D6 different phenotypes are substantially significant, as individuals with diminished CYP2D6 metabolic capacity cannot metabolize drugs as effectively as NMs. Consequently, PMs and IMs are prone to adverse effects and intoxication depending on the substrate. On the other hand, the accelerated pattern of metabolism in UMs can lead to therapeutic failure with the recommended drug dosing. The opposite is true in terms of prodrugs (such as codeine, tramadol, and tamoxifen) that oblige bioactivation, where UMs are prone to a higher risk of adverse effects and intoxication [32]. Moreover, the clinical relevance of IM phenotype is more evident when CYP2D6 substrates are concomitantly administered with CYP2D6 inhibitors. This phenomenon is known as phenoconversion, as drug interactions mimic the effect of inherited variations. For instance, when an individual genotyped as an IM receives a strong CYP2D6 inhibitor, such as fluoxetine and paroxetine, one's metabolic capacity will be similar to that of a PM [46]. Therefore, the Food and Drug Administration (FDA) marked the *CYP2D6* genotype as a pharmacogenomic biomarker in the labeling of numerous drugs [47]. Furthermore, CPIC has published to date six guidelines for drugs affected by *CYP2D6* genetic polymorphisms, which provide drug-dependent specific therapeutic recommendations based on *CYP2D6* genotype [48–53].

Pharmacogenetics is considered one of the pillars of individualized medicine [54]. Given the pivotal role of CYP2D6 in drug metabolism, determining the frequencies of *CYP2D6* alleles across world populations as well as identifying their impact on treatment outcomes is a critical step towards translating pharmacogenetic information into clinical settings and optimizing genotype-guided treatment. Studies of *CYP2D6* genetic polymorphisms are under-represented in quite a large number of Arab populations. Moreover, expanding the number of healthy individuals representing various Arab countries and investigating more alleles are needed to enrich the available information about the frequencies of *CYP2D6* alleles and broaden our knowledge of the genetic make-up of this unique ethnic group.

## Conclusions

Our study has proved uneven *CYP2D6* allelic frequencies across Arab populations. Considered together, active *CYP2D6* gene duplications, especially CYP2D6*2xN, presented at high frequency among Arabs compared to other ethnicities, whereas CYP2D6*41 was the most prevalent decreased function allele in the Arab populations, distinguishing them from other ethnicities where the reduced-function alleles were mostly corresponding to CYP2D6*10 in Asians or CYP2D6*17 and CYP2D6*29 in Sub-Saharan Africans and African-Americans/Afro-Caribbeans. Among non-functional alleles, CYP2D6*4 was the most studied allele and reported at lower frequencies than the frequency in Europeans, while other null alleles were infrequent. However, our findings emphasize the need for consistency in genotype profiling by following the criteria and recommendations put forward by PharmVar, PharmGKB, and other key pharmacogenomics consortia and necessitate conducting further studies to better assess the prevalence of the different *CYP2D6* alleles across the 22 Arab countries, especially in countries that lack data on the frequency of *CYP2D6* genotypes.

## Data Availability

Not applicable.

## Abbreviations

ACS: Acute coronary syndrome
AS: Activity score
CNVs: Copy number variations
CPIC: The Clinical Pharmacogenetics Implementation Consortium
CYP2D6: Cytochrome P450 2D6
DPWG: The Dutch Pharmacogenetics Working Group
FDA: Food and Drug Administration
IMs: Intermediate metabolizers
MENA: Middle East and North Africa
NMs: Normal metabolizers
OP: Organophosphate
PharmGKB: The Pharmacogenomics Knowledge Base
PCR–RFLP: Polymerase chain reaction-restriction fragment length polymorphism
PMs: Poor metabolizers
SNPs: Single nucleotide polymorphisms
UAE: The United Arab Emirates
UMs: Ultrarapid metabolizers
